# Adolescent Suicide Ideation, Depression and Self-Esteem: Relationships to a New Measure of Gender Role Conflict

**DOI:** 10.3389/fpsyg.2020.00111

**Published:** 2020-02-21

**Authors:** Cormac O’Beaglaoich, Jessica McCutcheon, Paul F. Conway, Joan Hanafin, Todd G. Morrison

**Affiliations:** ^1^School of Education, University of Limerick, Limerick, Ireland; ^2^National Institute for Studies in Education, Limerick, Ireland; ^3^Department of Psychology, University of Saskatchewan, Saskatoon, SK, Canada; ^4^Inclusion in Education and Society Research Group, School of Education, Trinity College Dublin, Dublin, Ireland

**Keywords:** suicide ideation, boys, masculinity, gender role conflict, Ireland, adolescence

## Abstract

Among 15–24 year olds in Ireland, completed suicide was responsible for 4.1 times more male deaths than female deaths in 2014 (World Health Organization [WHO], 2017). Few international research studies have investigated the relationship between masculinity [as assessed by a measure of gender role conflict (GRC)] and suicide ideation, and none have done so with Irish adolescents. Therefore, the purpose of the current study was to investigate the relationships between a new measure of GRC developed specifically for use with Irish adolescents (I-GRCS-A; O’Beaglaoich et al., 2016), and depression, self-esteem, and negative/protective suicide ideation. A sample of 176 adolescent boys (*M* = 16.9, *SD* = 0.94) from a non-clinical population participated in the study. Regression analyses and tests of mediation revealed that depression significantly mediated the relationship between GRC and negative suicide ideation, whilst self-esteem and depression significantly mediated the relationship between GRC and positive suicide ideation. Implications and limitations of the current study are outlined and directions for future research are discussed.

## Introduction

Official statistics provided by the World Health Organization [WHO] (2014a) indicated that 1.4% of all global deaths (803,900 persons) in 2012 occurred via suicide^1^. While these are global statistics, suicide is categorized as the fifteenth highest cause of death worldwide and approximately 75% of these suicides had occurred in middle and low-income countries (World Health Organization [WHO], 2014b). Cerel et al. (2018) estimated that, for a single suicide, an average of 135 people knew the person in question, resulting in a large circle of people (potentially) needing clinical services or support. Males are almost twice as likely to die by completed suicide compared to females^2^ (World Health Organization [WHO], 2014a) and, in western countries, males die by suicide three to four times more often than do females (Värnik, 2012), indicating that factors associated with gender relate to increased prevalence of completed suicides.

Adolescent suicide has emerged as a critical public health concern (e.g., Beautrais, 2000; Gould et al., 2003; Bridge et al., 2006; Hinduja and Patchin, 2018; Toomey et al., 2018) and is the second highest cause of death among young adolescents and young adults (15–29 year olds; World Health Organization [WHO], 2014a). The high incidence of adolescent suicide is believed to be precipitated by the myriad societal stressors experienced by young people (Turner et al., 2002). To illustrate: the following variables have been associated with suicide among adolescents: (a) substance use and/or abuse (e.g., Beautrais, 2000; Brennan and McGilloway, 2012); (b) affective mood disorders (e.g., Beautrais, 2000; Balázs et al., 2013; (c) antisocial behavior (e.g., Beautrais, 2000; Linker et al., 2012); (d) lower self-esteem (e.g., Overholser et al., 1995; Garber et al., 1997); (e) medical illnesses such as stroke and chronic illness (e.g., Ferro et al., 2017; Kim and Lee, 2018), and (f) depression (Lee et al., 2006). Other factors that have been associated with adolescent suicide include gender identity (e.g., Skagerberg et al., 2013; Yadegarfard et al., 2014), sexual orientation (e.g., Beautrais, 2000; Baams et al., 2015) and socioeconomic disadvantage (Yildiz et al., 2018).

In Ireland, specifically, data from the Central Statistics Office [CSO] (2014) and World Health Organization [WHO] (2014b) reveal that, from 2000 to 2012, suicide rates have consistently been over three times higher for males compared to females. This difference persists across all age groups. Focusing more narrowly on 15–24 year olds in Ireland, suicide was responsible for 3.7 times more male deaths than female deaths in 2012 (World Health Organization [WHO], 2014c) and 4.1 times more male deaths in 2017 (World Health Organization [WHO], 2017). Suicide also has persisted as the leading cause of death for males in this age range (Lynch et al., 2006). To put this into perspective, suicide was responsible for more deaths in 2012 among 15–24 year old Irish males than cancer, traffic accidents, and heart, liver, and kidney disease combined (World Health Organization [WHO], 2014c).

There is a dearth of research exploring adolescent suicidality within an Irish context; however, reviewing the available literature reveals that gender differences exist among suicide risk factors for Irish adolescents. For example, in 2003/2004, on the basis of cross-sectional survey data (*N* = 3,631) collected from 39 second-level schools in counties Kerry and Cork, and in conjunction with national suicide rates at the time, McMahon et al. (2014) reported that males were more likely than females to engage in *fatal* self-harm (i.e., males who engaged in self-harm possessed an especially high risk of *completing* suicide). This research indicates that Irish males use more lethal methods of suicide (i.e., 90% of adolescent males used hanging as their primary method of suicide) compared to females (i.e., 60% of females used overdose as their primary method of suicide) making completion more likely to occur with less opportunity for last minute intervention.

Using data from the same survey in counties Kerry and Cork, McMahon et al. (2010) examined various factors related to deliberate self-harm (DSH; i.e., a precursor to suicide) for males and females aged 15–17 (*N* = 3,801). Factors such as drug use, smoking, drinking, and knowing a family member or a friend who had engaged in DSH were found to have associations with DSH for both males and females. For females, interpersonal relationship factors (i.e., fights with parents, difficulty making/keeping friends, forced sexual activity, DSH engaged in by a family member, and lower self-esteem) were significantly related to DSH. Conversely, school-related problems (i.e., problems with schoolwork, being bullied) and psychological factors (i.e., anxiety and impulsivity) emerged as significantly associated with DSH for males but not females. Further, concerns regarding sexual orientation and being bullied at school were more strongly associated with DSH for males.

Given these risk factors for young Irish males, it is surprising that the relationship between suicidality and masculinity [in particular, gender role conflict (GRC)] has received little empirical attention.

### Gender Role Conflict

Gender role conflict is defined as “a psychological state in which the socialized male gender role has negative consequences for the person and others” and occurs when “rigid, sexist, or limiting gender roles result in restriction, devaluation, or violation of self and/or others” (O’Neil, 2008, p. 362). With over 250 studies conducted to date, GRC has been shown to be associated with numerous psychological health indicators. O’Neil’s (2008) theory proposes three components that cause GRC: (1) gender role devaluations (i.e., negative assessment of self or others when individuals conform to, or deviate from, the attributes characteristic of traditional or hegemonic masculinity); (2) gender role restrictions (i.e., limiting self or others to stereotypic norms of masculinity); and (3) gender role violations (i.e., when people hurt themselves or others [or are hurt by others] when deviating from or conforming to the norms associated with hegemonic masculinity). There is variation in the degree to which males endorse specific aspects of prescribed masculinity (O’Neil et al., 1986). Further diversity in the variations of experienced GRC is dependent on developmental stages, cultural and cohort specific definitions of masculinity and gender role stereotypes (Kahn, 2009).

The most widely used measure of GRC is the Gender Role Conflict Scale (GRCS; O’Neil et al., 1986), which is regarded as the “most well-known instrument within the traditional counseling literature” focusing on masculinity (Betz and Fitzgerald, 1993, p. 360). Previous research attests to the psychometric soundness of the GRCS (e.g., Thompson and Pleck, 1986; Eisler and Skidmore, 1987; Levant et al., 1992; Fischer and Good, 1997; O’Neil, 2008). The GRCS also has made “an important contribution to men’s health research,” with “11 out of 13 studies reviewed by O’Neil (2008) document[ing] a negative correlation between GRC and self-esteem, 12 out of 15 studies report[ing] a positive correlation between GRC and anxiety, and 24 out of 27 studies [finding] positive correlations between GRC and depression” (O’Beaglaoich et al., 2013, p. 17).

Blazina et al. (2005) created an adolescent permutation of the GRCS (GRCS-A). From the original scale (37-items), 29 items were retained across four equivalent factors although three were renamed to better capture the latent construct captured by the items [i.e., the “Conflict Between Work and Family Relations” subscale was named “Conflict Between Work, School and Family” (CBWSF); “Success, Power and Competition” was relabeled “Need for Success and Achievement” (NSA); and the “Restricted Affectionate Behavior Between Men” subscale was labeled “Restricted Affection Between Men” (RAM)]. Cronbach’s alpha (i.e., scale score reliability coefficients) ranged from 0.70 to 0.82 and test–retest reliability scores varied from 0.60 to 0.95 (O’Neil, 2008). The GRCS-A correlated strongly with the original version of the scale (i.e., the GRCS, *r* = 0.88); however, this correlation may be inflated due to the shared error of items in the short and long versions being correlated twice (Smith et al., 2000). The GRCS-A also correlated with scores on another scale assessing hegemonic masculinity [the Male Role Attitude Scale (MRAS), *r* = 0.37].

O’Beaglaoich et al. (2013) outlined a number of criticisms of both the GRCS and the GCRS-A. O’Beaglaoich et al. (2015a) subsequently tested the psychometric properties of the GRCS-A with a sample of Irish adolescents and found that the measure was not optimal for distribution to this group (e.g., confirmatory factor analyses revealed that various items contributed to model misfit). The researchers posited that their findings “may reflect different constellations of masculinity within an Irish adolescent sample or, possibly, the use of wording that was ill-suited for this group of young boys” (p. 38). Despite supporting the general tenets of GRC theory, qualitative investigations of the items on the GRCS-A, conducted with another sample of Irish adolescents, revealed that numerous items were problematic. For example, the statement, “Verbally expressing my love to another man is hard for me” was regarded as suitable only for girls or gay men (i.e., straight boys don’t “love” other boys). The phrase “hard for me” also triggered laughter as one sample of boys associated the word “hard” with getting an erection. Based on focus groups and personal interviews with Irish boys, O’Beaglaoich et al. (2013) found that nine of the items on the GRCS-A were regarded as unsuitable.

In response to the aforementioned concerns, O’Beaglaoich et al. (2016) published a study detailing a new measure of GRC for use within Ireland (I-GRCS-A). Scale items were generated from four themes identified through qualitative interviews with Irish boys (O’Beaglaoich et al., 2015b). These items were subsequently validated by content and lay experts and exploratory factor analysis identified a unidimensional structure.

As detailed above, the GRC research paradigm has repeatedly shown correlations between scores on the GRCS and a number of indicators of poor physical and psychological wellbeing (e.g., O’Neil, 2008); however, a literature search yielded only two published studies that investigated the association between GRC and suicide in adolescent/young adult populations.

Galligan et al. (2010) explored the relationship between GRC and suicidality in young adult males within a resilience paradigm. Specifically, undergraduate male participants (*N* = 362) were asked to complete a questionnaire package containing items that measured GRC, resilience, sexual orientation, and basic demographics. Results indicated that participants’ level of restrictive emotionality, which is one facet of GRC and denotes an inability to disclose or anxiety surrounding the disclosure of feelings and emotions, was inversely associated with positive identity asset resilience (i.e., an individual’s positive sense of self). The authors link the absence of this form of resilience with literature suggesting such deficits are indicative of a greater propensity for depression and subsequent suicide.

Among a large sample of American adolescents (*N* = 2,189; 58.3% male; 13–18 years old), Jacobson et al. (2011) found that restrictive emotionality correlated positively with depressive symptoms and suicide ideation/intent. Further, those obtaining higher scores on a measure of restrictive emotionality were 11 times more likely to report elevated depression scores; three times more likely to report suicide ideation (after controlling for depressive symptoms); and more than twice as likely to report attempting suicide (again, after controlling for depressive symptoms). These researchers did not examine the association between the other GRCS-A factors (e.g., NSA) and suicide ideation/intent. Thus, it is unknown how these other facets of GRC link with both depression and indicators of suicidality.

Using a measure of GRC that is appropriate for adolescents outside of a North American context is important. It is equally important, however, that researchers be cognizant of the different ways in which suicide ideation can be assessed.

### Measuring Suicidal Ideation

Osman et al. (2003) note that, traditionally, much suicidality research has focused on negative risk factors (i.e., conditions or attributes that influence an individual to engage in self-harm behaviors). One risk factor that has received considerable attention is depression which has been identified as one of the primary predictors of suicide ideation (Harlow et al., 1986; De Man and Leduc, 1995; Lee et al., 2006). In addition, low self-esteem accounts for variance in suicide ideation beyond that accounted for by depression (Overholser et al., 1995). A limitation common of many indicators of suicide ideation is their tendency to focus on negative risk factors; a focus that precludes these measures from capturing the multifaceted nature of suicide ideation (Osman et al., 2003). For example, harms-based measures overlook the fact that suicide ideation also may involve *protective* factors (i.e., individuals evidencing problematic levels of risk may still possess an innate desire to live which can, ultimately, prevent them from engaging in self-harm behaviors; Osman et al., 2003). Therefore, scales that capture *both* protective and risk factors simultaneously would appear to offer the most comprehensive assessment of suicide ideation.

To fulfill this objective, Osman et al. (1998) developed the Positive and Negative Suicide Ideation Inventory (PANSI). The PANSI is a 14-item self-report scale that consists of two factor-analytically derived subscales: Negative Suicide Ideation (PANSI-NSI; eight items) and Positive Ideation (PANSI-PI; six items). Participants are asked to provide the frequency with which they experienced each item over the course of the past 2 weeks. Since each subscale measures opposing constructs of suicide ideation, separate scores are computed rather than a total score. Higher scores on the PANSI-NSI indicate greater negative suicide ideation while higher scores on the PANSI-PI represent greater positive ideation (e.g., individuals with higher scores on the PANSI-NSI and lower scores on the PANSI-PI would be deemed as being at greater risk of engaging in self-harm behaviors). Initially validated using an adult population, the PANSI was subsequently found to be psychometrically sound with both inpatient (Osman et al., 2002) and community (Osman et al., 2003) adolescent samples. The PANSI has been used with adolescents in China (e.g., Chang et al., 2009), Thailand (e.g., Yadegarfard et al., 2013), Malaysia (Ling and Yaacob, 2015; Sinniah et al., 2015), and Pakistan (Yasien and Ahmad, 2015). Employing the PANSI provides researchers with more robust data regarding the multifaceted construct of suicide ideation.

## Present Study

Within an Irish context, no study has investigated the relationship between GRC and suicide ideation, and no study has investigated the GRC/suicidality relationship among those at greatest risk of committing suicide (i.e., male individuals between the ages of 15–24). The purpose of the current study, therefore, was to address this omission by investigating the relationship between the GRC and suicide ideation among a non-clinical sample of Irish adolescent males.

Depression and self-esteem are well established predictors of suicide ideation and past research within the GRC paradigm has found significant correlations between GRC and self-esteem as well as depression (see O’Neil, 2008 for a review). O’Beaglaoich (2014) and O’Beaglaoich et al. (2016) reported that scores on the GRCS-IA, a measure of GRC designed for use with Irish boys, correlated negatively with a measure of self-esteem, and positively with a measure of depression. Thus, in the current study, it was predicted that similar associations would be observed (i.e., GRC would be inversely correlated with self-esteem [H1] and positively correlated with depression [H2]). Social scientists have not investigated whether the association between GRC and indices of suicide ideation are mediated by depression and self-esteem; thus, no formal hypotheses in terms of mediation were generated.

## Materials and Methods

### Participants

A sample of 176 boys aged 15–20 (*M* = 16.9, *SD* = 0.94), from a non-clinical population, situated in six schools in Ireland took part in this study. Participants self-identified as fourth (*n* = 21; 11.9%), fifth (*n* = 64; 36.4%), and sixth (*n* = 91; 51.7%) year students and all attended either a mixed or single-sex secondary school in Ireland. A majority (*n* = 154, 87.5%) reported being “Irish,” while 7.4% (*n* = 13) selected “any other white background,” and eight participants (4.5%) reported being of “any other Asian background.” One participant (0.6%) did not report their ethnicity. Approximately 86.9% (*n* = 153) self-identified as “exclusively heterosexual,” followed by 5.7% (*n* = 10) identifying as “more heterosexual than gay,” 2.8% (*n* = 5) as “bisexual,” and 1.7% (*n* = 3) each as “more gay than heterosexual” and “exclusively gay.” Two participants (1.1%) did not report their sexual orientation.

### Measures

#### Depression

The Center for Epidemiologic Studies Depression Scale (CES-D; Radlof, 1977) consists of 20 items and has been used extensively in non-clinical samples of adults, adolescents, and children for the screening of depression. Responses are provided using a four point Likert scale ranging from one (Rarely or none of the time) to four (Most or all of the time) to indicate how often each item was experienced in the previous week. Scores can range from 20 to 80, with scores of 36 or higher indicating the possible presence of depression. A sample item reads “I felt that everything I did was an effort.” Previous research has demonstrated that the CES-D possesses sound psychometric properties (e.g., Garrison et al., 1991). For the present sample, Cronbach’s alpha was 0.92 (95% CI = 0.90, −0.94), indicating good scale score reliability.

#### Socio Economic Status

The Family Affluence Scale II (FAS II; Boyce and Dallago, 2004) is a four item scale designed as an alternative measure of family wealth for adolescent populations. The FAS II employs a mixed response option: (1) a dichotomous (No [1]/Yes [2]) option for one item (i.e., “Do you have your own bedroom for yourself?”); (2) a three point (No [1]/Yes, one [2]/Yes, two or more [3]) option for one item (i.e., “How many vehicles in your family home?”); (3) a four point (Not at all [1]/Once [2]/Twice [3]/More than twice [4]) option for one item (i.e., “During the past 12 months, how many times did you travel away on holiday with your family?”); and (4) a separate four point (None [1]/One [2]/Two [3]/More than two [4]) response format for one item (i.e., “How many computers does your family own?”). Family affluence is calculated using a three point ordinal scale, where scores 4–6 indicate low affluence; 7–9 denote middle affluence, and 10–13 suggest high affluence. Since Boyce et al. (2006) report that scale score reliability is “not a prerequisite for formative indexes such as the FAS” (p. 481), Cronbach’s alpha was not calculated. Past suicide research suggests that populations with low and middle socioeconomic status (SES) are at greater risk for suicide than more affluent status groups (Ko et al., 2014).

#### Gender Role Conflict

Previous research has called into question the psychometric properties of the original GRCS-A (O’Beaglaoich et al., 2015a, b). As a result, O’Beaglaoich (2014) developed the nine item unidimensional Irish Gender Role Conflict Scale for Adolescents (I-GRCS-A). A five-point Likert scale, ranging from one (Never) to five (Almost Always), is used to indicate the frequency with which a respondent experiences each of the items. Scores can range from 9 to 45, with higher scores indicating greater GRC. A sample item reads “It bothers me that lads expect you to be good craic [fun] even when you’re in bad form.” O’Beaglaoich et al. (2016) reported sound psychometric properties for the I-GRCS-A. For the current sample, Cronbach’s alpha was 0.82 (95% CI = 0.78, −0.86) indicating good scale score reliability.

#### Suicide Ideation

Suicide ideation was measured using the Positive and Negative Suicide Ideation Scale (PANSI; Osman et al., 1998). As mentioned, the PANSI is a 14-item two-dimensional self-report screening instrument that assesses the frequency of negative risk (PANSI-NSI; 8 items) and protective factors (PANSI-PI; 6 items) related to suicide behavior. The time frame for rating PANSI items is “the past 2 weeks, including today.” Each item is rated on a five-point Likert scale ranging from one (None of the time) to five (Most of the time). Scores on the PANSI-PI can range from 6 to 30, with higher scores indicating lower suicidal ideation. For the PANSI-NSI, scores can range from 8 to 40, with higher scores indicating greater suicidal ideation. A sample item from the PANSI-PI is “Felt hopeful about the future because things were working out for you?” and a sample item from the PANSI-NSI is “Seriously considered killing yourself because you could not live up to the expectations of other people?” Previous research has demonstrated the PANSI possesses good psychometric properties (e.g., Osman et al., 2002). For the present sample, Cronbach’s alpha coefficients of 0.95 (95% CI = 0.94, −0.96) and 0.82 (95% CI = 0.78, −0.86) were obtained for the PANSI-NSI and PANSI-PI, respectively.

#### Self-Esteem

The Rosenberg Self-Esteem Scale (RSES; Rosenberg, 1965) is a 10 item trait measure of global self-esteem. Responses are provided using a four-point Likert scale ranging from one (Not at all) to four (Very much so). Total scores range from 10 to 40, with higher scores indicating greater self-esteem. A sample item reads “I take a positive attitude toward myself.” Previous research has demonstrated that the RSES possesses sound psychometric properties (e.g., Mullan and NicGabhainn, 2002). For the present sample, Cronbach’s alpha was 0.86 (95% CI = 0.82, −0.89) indicating good scale score reliability.

### Procedure

Ethical approval was obtained by the Research Ethics Committee (REC) at the National University of Ireland, Galway. Forty-three schools were contacted at random from a list of schools provided by the Department of Education. Of the 43 schools that were contacted, two schools agreed to take part in the research. Schools were contacted initially by a letter sent to the Principal/Board of Management and then by telephone. Two hundred and fifty consent forms were administered to School A and 197 consent forms to School B. Following approval from the Board of Management of the schools, the guidance counselors in each school co-ordinated and oversaw the distribution and completion of the surveys. Posters for support agencies (i.e., Aware, Spunout.ie and the Samaritans) and specially designed business cards were put up on the schools’ noticeboards. Consent forms were distributed to boys during class time and participants were asked to bring the information sheets and consent forms to their parent(s)/guardian(s). In line with REC guidelines, active parental consent was solicited. Participants whose parents wanted their child to take part in the research were asked to bring the signed consent form to the guidance counselor within a 2 week window. During this period, guidance counselors also reminded potential participants to bring the consent forms home to be signed. The numbers of consent forms returned were: 170 (School A; 68% return) and 41 (School B; 21% return). While speculative, it is possible that the difference in return rates may be attributable to a highly supportive and proactive guidance counselor affiliated with School A.

Due to tragic circumstances (i.e., the suicide of a student attending School A), administration of the questionnaire was canceled at that school. Consequently, 48 additional schools were contacted to partake in the research. Five schools agreed to take part in the study bringing the total number of schools to six.

The response rates were: School B (30 out of 42 students returning the consent forms participated); School C (40 out of 47; 94 forms distributed initially); School D (36 out of 53; 150 forms distributed]); School E (18 out of 42; 110 forms distributed); School F (11 out of 22; 80 forms distributed); and, finally, School G (41 out of 47; 100 consent forms distributed).

Two weeks after the consent forms had been distributed, the questionnaire was administered to students. Respondents that did not return signed parental/guardian consent forms were unable to participate. The questionnaire, which was presented to participants in an A4 sealable envelope, consisted of demographic items and the measures of GRC, self-esteem, depression and suicidal ideation. The information sheet and consent form were included as was a business card listing contact details of relevant support agencies. The questionnaire took approximately 10–15 min to complete.

## Results

The data were initially screened for missing values. Little’s missing completely at random (MCAR) test was conducted and was found to be statistically significant (χ^2^ [1343] = 1528.704, *p* < 0.001) suggesting the data were *not* MCAR. The highest level of “missingness” was determined to be 2.8%; therefore, the expectation maximization (EM) algorithm for imputing missing data was employed (For additional details about EM, see Fish et al., 2013).

After the missing values were replaced using EM, descriptive statistics were computed. Total CES-D scores ranged from 20 to 77. On average, participants scored slightly below the cut-off score of 36 (*M* = 34.73; *SD* = 11.36) suggesting an overall absence of depression among boys in the sample. However, 35.2% (*n* = 62) of participants did meet the depression criterion of a score of 36 or higher. In regards to self-esteem, total scores ranged from 10 to 40; however, the mean score was above the scale midpoint (*M* = 30.99; *SD* = 6.32). Likewise, although GRC scores ranged from 9 to 45, the mean score was 25.98 (*SD* = 7.33), which is slightly below the scale’s midpoint. Scores on the FAS II ranged from 5 to 13, with a mean score of 9.85 (*SD* = 1.50), suggesting that participants were fairly affluent. Mean scores on the PANSI-NSI (*M* = 11.02; *SD* = 5.80) and the PANSI-PI (*M* = 22.86; *SD* = 4.71) subscales suggest that participants had few risk factors for suicide and many protective factors, respectively. To illustrate, 56.8% (*n* = 100) of respondents obtained a score of 8 on the PANSI-NSI subscale, which is the lowest possible score. More detailed information about the distribution of responses on the PANSI subscales can be found in Table 1.

**TABLE 1:** Distribution of scores on the PANSI-NSI and PANSI-PI.

Item	Responses options				
	
	None of the time	Rarely	Sometimes	Often	Most of the time
**PANSI-NSI**					
Seriously considered killing yourself because you could not live up to the expectations of other people?	76.1% (*n* = 134)	14.8% (*n* = 26)	6.8% (*n* = 12)	0.6% (*n* = 1)	1.1% (*n* = 2)
Felt hopeless about the future and you wondered if you should kill yourself?	72.2% (*n* = 127)	14.2% (*n* = 25)	9.7% (*n* = 17)	1.7% (*n* = 3)	2.3% (*n* = 4)
Felt so unhappy about your relationship with someone you wished you were dead?	75.0% (*n* = 132)	11.4% (*n* = 20)	8.5% (*n* = 15)	2.3% (*n* = 4)	2.3% (*n* = 4)
Thought about killing yourself because you could not accomplish something important in your life?	80.1% (*n* = 141)	8.5% (*n* = 15)	6.8% (*n* = 12)	2.8% (*n* = 5)	1.7% (*n* = 3)
Thought about killing yourself because you could not find a solution to a personal problem?	79.0% (*n* = 139)	11.4% (*n* = 20)	7.4% (*n* = 13)	1.7% (*n* = 3)	0.6% (*n* = 1)
Thought about killing yourself because you felt like a failure in life?	77.8% (*n* = 137)	13.1% (*n* = 23)	4.5% (*n* = 8)	2.3% (*n* = 4)	2.3% (*n* = 4)
Thought that your problems were so overwhelming that suicide was seen as the only option for you?	79.0% (*n* = 139)	6.8% (*n* = 12)	10.2% (*n* = 18)	1.7% (*n* = 3)	1.7% (*n* = 3)
Felt so lonely or sad you wanted to kill yourself so that you could end your pain?	77.3% (*n* = 136)	14.8% (*n* = 26)	4.0% (*n* = 7)	2.3% (*n* = 4)	1.7% (*n* = 3)
**PANSI-PI**					
Felt that you were in control of most situations in your life?	2.3% (*n* = 4)	11.4% (*n* = 20)	23.3% (*n* = 41)	37.5% (*n* = 66)	25.6% (*n* = 45)
Felt hopeful about the future because things were working out well for you?	4.5% (*n* = 8)	7.4% (*n* = 13)	19.9% (*n* = 35)	31.3% (*n* = 55)	36.9% (*n* = 65)
Felt excited because you were doing well at school or at work?	5.7% (*n* = 10)	8.5% (*n* = 15)	36.9% (*n* = 65)	35.8% (*n* = 63)	13.1% (*n* = 23)
Felt confident about your ability to cope with most of the problems in your life?	5.7% (*n* = 10)	7.4% (*n* = 13)	22.7% (*n* = 40)	36.9% (*n* = 65)	26.7% (*n* = 47)
Felt that life was worth living?	3.4% (*n* = 6)	5.1% (*n* = 9)	8.5% (*n* = 15)	19.3% (*n* = 34)	63.6%(*n* = 112)
Felt confident about your plans for the future?	3.4% (*n* = 6)	11.4% (*n* = 20)	22.7% (*n* = 40)	31.8% (*n* = 56)	30.7% (*n* = 54)

In addition to descriptive statistics, intercorrelations among scale scores were examined (see Table 2). With the exception of family affluence, all measures were found to significantly correlate with one another at a moderate-to-strong level. Those who evidenced higher depressive scores also had higher levels of GRC (*r* = 0.55, *p* < 0.01), greater suicide risk factors (*r* = 0.70, *p* < 0.01), fewer suicide protective factors (*r* = −0.75, *p* < 0.01), and lower self-esteem (*r* = −0.76, *p* < 0.01). Individuals with greater GRC also had fewer protective (*r* = −0.40, *p* < 0.01) and greater risk factors for suicide (*r* = 0.43, *p* < 0.01), and had lower self-esteem (*r* = −0.49, *p* < 0.01). Greater self-esteem was associated with greater protective (*r* = 0.77, *p* < 0.01) and fewer risk factors for suicide (*r* = −0.62, *p* < 0.01). The two PANSI subscales were negatively correlated, with greater protective factors being associated with fewer risk factors (*r* = −0.67, *p* < 0.01).

**TABLE 2:** Intercorrelations among scale scores.

Measures	CES-D	FAS II	I-GRCS-A	PANSI-NSI	PANSI-PI	RSES
CES-D	–					
FAS II	–0.069	–				
I-GRCS-A	0.551**	–0.005	–			
PANSI-NSI	0.703**	0.055	0.434**	–		
PANSI-PI	−0.748**	0.115	−0.401**	−0.670**	–	
RSES	−0.756**	0.071	−0.487**	−0.620**	0.769**	–

**p < 0.01. CES-D = The Center for Epidemiologic Studies Depression Scale; FAS II = Family Affluence Scale; I-GRCS-A = The Irish Gender Role Conflict Scale for Adolescents. RSES = Rosenberg Self-Esteem Scale; PANSI-Positive = Positive Suicide Ideation; PANSI-Negative = Negative Suicide Ideation.

To assess whether the relationship between GRC and suicide ideation was mediated by depression and self-esteem, two mediation analyses were conducted: one for the PANSI-NSI subscale and one for the PANSI-PI subscale. The PANSI-PI subscale met the assumptions needed to conduct the regression analysis; however, due to the significant negative skewness in the PANSI-NSI data, the assumptions of normality and homoscedasticity were violated. The data were then transformed, but as doing so did not yield significant improvements in normality, the untransformed data were employed. To mitigate the effects of the data violations, we used bootstrapping (Preacher and Hayes, 2004), which has been found to be more robust to violations of distributional assumptions (Yuan and MacKinnon, 2014).

**FIGURE 1:**
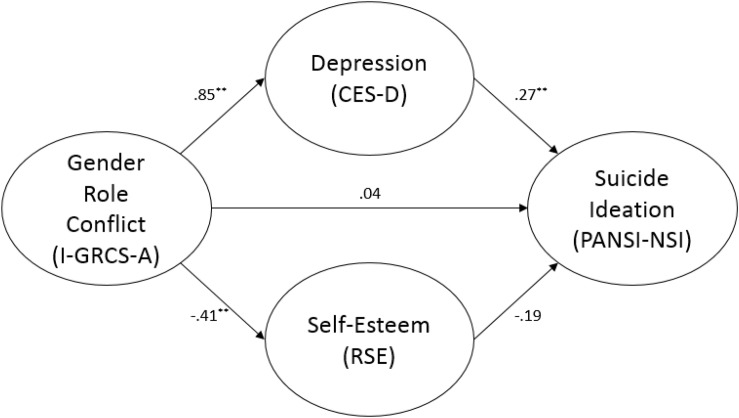
Mediation analysis with PANSI-NSI. ^∗∗^ *p* < 0.01.

The relationship between GRC and negative suicide ideation was fully mediated by depression scores. As Figure 1 illustrates, GRC was a significant predictor of both depression (*b* = 0.848, SE = 0.126, *p* < 0.001) and self-esteem (*b* = −0.411, SE = 0.071, *p* < 0.001). However, only depression significantly predicted negative suicide ideation (*b* = 0.274, SE = 0.067, *p* < 0.001). On its own, GRC was not significantly associated with negative suicide ideation (*b* = 0.038, SE = 0.046, *ns*). Approximately 72% of the variance in negative suicide ideation was accounted for by the predictors (*R*^2^ = 0.717). The indirect effect was tested using a bootstrap estimation approach with 5000 samples. The results revealed the indirect coefficient for GRC was statistically significant, *b* = 0.232, SE = 0.072, *p* < 0.001, 95% CI = 0.114, 0.403.

**FIGURE 2:**
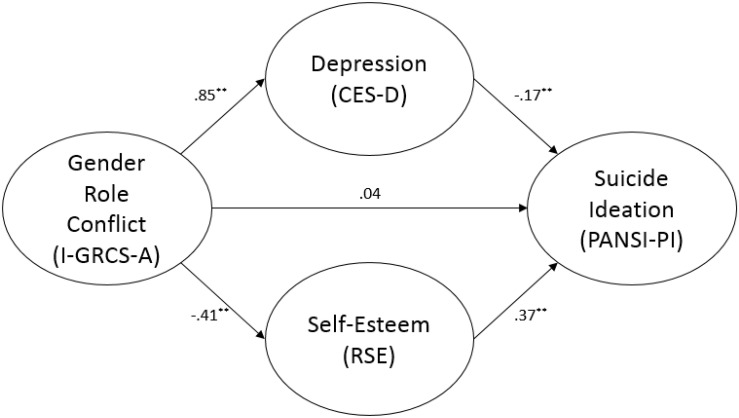
Mediation analysis with PANSI-PI. ^∗∗^ *p* < 0.01.

The second mediation analysis indicated that the relationship between GRC and positive suicide ideation was fully mediated by both depression and self-esteem (see Figure 2). Depression (*b* = −0.174, SE = 0.030, *p* < 0.001) and self-esteem (*b* = 0.371, SE = 0.057, *p* < 0.001) significantly predicted positive suicide ideation. Approximately 81% of the variance in positive suicide ideation was accounted for by the predictors (*R*^2^ = 0.812). Again, the indirect effect was tested using a bootstrap estimation approach with 5000 samples. The results showed that the indirect coefficients of GRC for both depression, *b* = −0.148, SE = 0.033, *p* < 0.001, 95% CI = −0.218, −0.091, as well as self-esteem, *b* = −0.152, SE = 0.034, *p* < 0.001, 95% CI = −0.223, −0.093, were statistically significant.

## Discussion

In the first study to examine the direct and indirect relationships between GRC and negative suicide and protective suicide ideation among a large sample from a non-clinical population of Irish adolescent boys, the results indicated that, while GRC correlates significantly with both factors protecting against and contributing to suicide ideation, these associations become statistically non-significant when depression and self-esteem are taken into consideration. Consistent with previous research, the mediation models revealed that depression correlated in the anticipated directions with both indices of suicide ideation whereas self-esteem correlated significantly with positive suicide ideation only. Boys who reported greater degrees of GRC were more likely to experience depression which, in turn, increased their likelihood of reporting negative suicide ideation. In contrast, boys who reported lower levels of GRC were less likely to experience depression and more likely to experience greater levels of self-esteem both of which appeared to increase boys’ reporting of protective factors that mitigated against suicide ideation, as determined by scores on the PANSI-PI.

Our findings support Houle et al.’s (2008) study with adult males which concluded that GRC is associated with variables that, in turn, may play a role in suicidality. Specifically, we found that scores on the I-GRCS-A correlated moderately with depression, a variable which – in turn – was linked with negative suicide ideation (i.e., depression fully mediated the association between GRC and scores on the PANSI-NSI). It is important to note that the current study goes beyond Houle et al.’s work by investigating the relationship between self-esteem, depression and suicidal ideation with a non-clinical population of adolescent boys. Using a “normal” sample of adolescent males, we obtained tentative support for a key tenet of GRC theory; namely, that restrictive gender roles can have negative health consequences for males.

Approximately 72% of the variance in negative suicide ideation was accounted for by depression, self-esteem (*ns*) and GRC and the indirect effect of GRC on negative suicide was statistically significant (i.e., *b* = 0.232, SE = 0.072, *p* < 0.001, 95% CI = 0.114, 0.403). This finding highlights that some boys are negatively affected by societal expectations and pressures placed on males in Irish society and these boys have an increased likelihood of experiencing depression which, in turn, is associated with an increased likelihood of experiencing negative suicide ideation. The finding that GRC role conflict correlated significantly with depression is consistent with studies conducted with adult men [e.g., 24 out of 27 studies found positive correlations between GRC and depression (O’Neil, 2008)].

As discussed previously, suicide appears to be a gendered problem with researchers identifying myriad contributing biological, psychological and societal factors that place males at greater risk. From a biological/hormonal stance, the hormone oxytocin, which seems to function as a facilitator for the affective empathic system, tends to be more present in females (Rueckert and Naybar, 2008). Lower levels of oxytocin in males may relate to why men, on average, experience more alexithymia (i.e., an inability to identify and describe emotions in the self; Levant et al., 2009). Further, males are more likely to respond to negative emotional states by externalizing depressive behaviors (Rice et al., 2013) and, on average, are more likely than women to: (a) use, misuse, or abuse alcohol or substances, especially as they relate to decreased inhibition (Russell et al., 2004); (b) engage in aggressive behavior (Hyde, 2005); (c) take risks; (d) be impulsive (Chapple and Johnson, 2007); and (e) isolate themselves from the support available from the greater social network (Eckersley and Dear, 2002). Additionally, some boys expressed that a general negative societal opinion of boys exists and is a cause of stress for them (i.e., an expectation that boys will misbehave; O’Beaglaoich et al., 2015b).

The externalizing symptoms above, combined with a lack of coping skills (or dysfunctional coping skills) exhibited by a proportion of males (Tamres et al., 2002), are hypothesized to occur in those who are unable and/or reluctant to seek assistance for personal problems (e.g., Russell et al., 2004) out of fear of being viewed as weak, inferior or vulnerable (e.g., Rochlen et al., 2010; Rice et al., 2013). These factors are shared correlates of both GRC (O’Neil, 2008; i.e., in particular the restrictive emotionality subscale of the GRCS) and suicide ideation. One explanation for the results of this study could be that young boys, who are less biologically predisposed (e.g., alexithymia and impulsivity) to cope with the expectations placed upon them as boys and who also have fewer relational and coping skills are more likely to express externalizing behaviors (e.g., depression indicators) and, in extreme cases, may be more likely to experience suicide ideation.

The I-GRCS-A was significantly related to self-esteem and depression, variables that were – in turn – significantly related to the PANSI-Positive subscale. Thus, the linkage between GRC and protective suicide ideation was fully mediated by both self-esteem and depression. Specifically, the “effects” of GRC on PANSI-Positive subscale scores appeared to be minimal when self-esteem and depression were taken into consideration. I-GRCS-A’s relationship with self-esteem is consistent with past research with Irish samples [e.g., *r* = −0.49 in this study; *r* = −0.45 (O’Beaglaoich et al., 2016)] and with past research using the adult version of the GRC scale (i.e., 11 out of 13 studies reviewed by O’Neil (2008) documented a negative correlation between GRC and self-esteem). Taken together, these correlations, which are congruent with O’Neil’s (2015) summary of the literature, underscore the important role that GRC seems to play in adolescent boys’ psychological well-being, as determined by self-esteem and depression.

Importantly, tests of mediation revealed that, while self-esteem did not significantly mediate the relationship between GRC and negative suicide ideation, it did serve as a mediator for the linkage between GRC and positive suicide ideation. These findings suggest that boys with lower levels of GRC may, in turn, have greater levels of self-esteem, with the latter construct potentially bolstering use of positive suicide ideation. This relationship is in line with past research; namely, that higher self-esteem appears to serve a protective function against suicidal ideation/attempts (De Man and Gutiérrez, 2002). Further research is needed to clarify why self-esteem only mediated the relationship between GRC and positive suicidality. However, one possible explanation resides in our use of the RSES to measure self-esteem. Mannarini (2010), for example, found that items on the RSES were differentially associated with measures of attachment as well as empathic and social self-efficacy. These forms of efficacy reflect (in order) one’s ability to “understand other people’s thoughts, feelings, and needs” (p. 231) and one’s skill at interpersonal relationships (e.g., making friends and performing in public). Specifically, using a sample of 435 Italian university students, Mannarini noted that items three and nine of the RSES were negatively related to empathic and social self-efficacy whereas item 1 was positively related to both types of self-efficacy. This differential pattern suggests that: (a) the wording of the items might affect participants’ responses; and (b) the wording of the items also might influence the omnibus sign of the association between global self-esteem, as measured by the total score on the RSES, and other variables. Thus, future tests of mediation using the RSES to assess self-esteem may benefit from examining each item on the RSES individually.

It should be noted that this sample was highly affluent. Past suicide research suggests that populations from low and middle SES are at greater risk for suicide than more affluent status groups; therefore, given the possibility of ceiling effects, it is not too surprising that family affluence did not correlate significantly with suicide ideation in this study. GRC and SES has only been examined in one study to date (Stillson et al., 1990) and the results indicated that men classified as low SES reported significantly more GRC role conflict than their higher SES counterparts. Also, as educational/occupational status increased, men’s GRC decreased (O’Neil, 2008). In relation to research on GRC, social scientists have proposed investigating the relationship between this construct and SES in adult samples (Liu et al., 2004; O’Neil, 2008); however, no empirical studies have been carried out to investigate the relationship and interactions between these variables for adolescents.

We recommend that researchers examine and build upon the theoretical explanations of the process by which GRC is related to depression and self-esteem. GRC theory offers explanations through a combination of biological, psychological and sociocultural factors; however, O’Neil (2015) states that men’s depression is “not fully understood or defined in gendered ways that enable clinicians to make effective interventions” (p. 16). Akin to what was noted for depression, no theoretical explanation has been offered which particularizes *how* GRC is related to or causes variations in self-esteem (O’Neil, 2008). Thus, we believe that refining theoretical propositions pertaining to GRC, ultimately, may assist in reducing the degree to which men strive to adhere to expectations surrounding hegemonic masculinity. Further, such reductions may play a role in efforts to enhance psychological wellbeing in boys.

Researchers could, in future, deconstruct known elements of GRC scales to see which constituent parts account for the greatest proportions of variance in depression as well as self-esteem. GRC is theorized to be experienced in three contexts: (a) within the self; (b) induced by others; and (c) expressed toward others (O’Neil, 1990). Thus, we recommend that specific measures be developed for each of these contexts as they relate to known components of GRC (e.g., restrictive emotionality). Developing measures that take these different contexts into account would enable researchers to determine whether certain instigators of GRC (e.g., self vs. others) account for more (or less) variance in measures of psychological wellbeing (O’Beaglaoich, 2014).

## Limitations

One limitation that is worth noting concerns the recruitment of participants. Finding schools that were willing to become involved in this research emerged as our biggest obstacle. Due to schools being over-burdened with workloads and the sensitive nature of the research topic, many of the institutions we targeted did not respond to our request or refused to take part. Three of the four schools had actively participated in government suicide prevention and educational strategies. Therefore, it is possible that, due to the fact that they attended “proactive” schools, the students participating in this research were better informed about suicide and/or were more likely to avail themselves of mental health supports in comparison to students from schools that did not participate. Further, an estimate of the boys whose parents refused to allow their children to take part was not recorded; thus, we were unable to perform demographic comparisons between boys that completed the survey and those that did not. One additional limitation is that, despite the unequivocal importance of using self-report measures to assess suicide ideation, there will always remain a proportion of ideations that are unexpressed due to lack of insight or reluctance (Wolk-Wasserman, 1986; Hennig et al., 1998; Palmieri et al., 2019).

## Conclusion

In conclusion, our findings suggest that, through its linkage with depression and self-esteem, GRC plays a significant role in the lives of adolescent boys’ mental health. Greater attention should be directed at understanding the ways in which boys navigate the demands of hegemonic masculinity and how their methods of navigation (potentially) impact their psychological health.

## Ethics Statement

This study was carried out in accordance with the recommendations of Ethical Guidelines at the National University of Ireland, Galway, with written informed consent from all subjects. All subjects gave written informed consent in accordance with American Psychological Association guidelines. The protocol was approved by the Ethics Committee at the National University of Ireland, Galway.

## Author Contributions

CO’B designed the study, applied for research ethics, collected and inputted the data, and analyzed and wrote portions of the manuscript. PC and JH wrote portions of the manuscript and provided feedback on all manuscript drafts. JM assisted with analyzing the data and provided feedback on all manuscript drafts. TM assisted with the design of the study, wrote portions of the manuscript, and provided feedback on all manuscript drafts.

## Conflict of Interest

The authors declare that the research was conducted in the absence of any commercial or financial relationships that could be construed as a potential conflict of interest.

## Footnotes

1 These statistics do not account for individuals who engaged in self-harm, had unsuccessfully attempted suicide, or experienced persistent suicidal ideation.

2 National suicide rates in 2012 in Bangladesh, China, Indonesia, Iraq, and Pakistan were slightly higher for females.
